# Imaging patterns of cerebral ischemia in hypereosinophilic syndrome: case report and systematic review

**DOI:** 10.1007/s10072-022-06134-4

**Published:** 2022-05-19

**Authors:** Kathrin Miethe, Elene Iordanishvili, Pardes Habib, Jens Panse, Stefan Krämer, Martin Wiesmann, Jörg B. Schulz, Omid Nikoubashman, Arno Reich, João Pinho

**Affiliations:** 1 Department of Neurology, University Hospital, RTWH Aachen University, Pauwelsstr. 30, 52074 Aachen, Germany; 2 Department of Diagnostic and Interventional Neuroradiology, University Hospital, RWTH Aachen University, Aachen, Germany; 3 JARA-BRAIN Institute Molecular Neuroscience and Neuroimaging, Forschungszentrum Jülich GmbH and RWTH Aachen University, Aachen, Germany; 4 Department of Hematology, Oncology, Hemostaseology, and Stem Cell Transplantation, University Hospital, RTWH Aachen University, Aachen, Germany; 5 Center for Integrated Oncology, Aachen Bonn Cologne Düsseldorf (CIO ABCD), Aachen, Germany; 6 Department of Nephrology and Clinical Immunology, University Hospital, RTWH Aachen University, Aachen, Germany

**Keywords:** Hypereosinophilic syndrome, Eosinophilia, Stroke, Magnetic resonance imaging

## Abstract

**Introduction:**

Ischemic stroke is a potential complication of hypereosinophilic syndromes (HES), and little is known about underlying pathophysiological mechanisms. We aimed to describe the imaging patterns of cerebral ischemia in patients with HES.

**Methods:**

An individual case is reported. A systematic PubMed review of all records reporting adult patients with HES who suffered ischemic stroke and for whom neuroimaging details of ischemic lesions were available was performed.

**Results:**

A 60-year-old man presented with progressive subacute gait difficulty and psychomotor slowing as well as an absolute eosinophilia (2.2 × 10^9^/L) at admission. Brain magnetic resonance tomography revealed multiple acute and subacute internal and external border zone infarcts. Cardiac diagnostic suggested the presence of endomyocarditis. After extensive diagnostic workup, idiopathic HES was diagnosed. The systematic review yielded 183 studies, of which 40 fulfilled the inclusion criteria: a total of 64 patients (31.3% female), with mean age 51.1 years and a median absolute eosinophile count at diagnosis of 10.2 × 10^9^/L were included in the analyses. A border zone pattern of cerebral ischemic lesions was reported in 41 patients (64.1%). Isolated peripheral infarcts were reported in 7 patients (10.9%). Sixteen patients had multiple acute infarcts with no border zone distribution (25.0%). An intracardiac thrombus was reported in 15/60 patients (25%), and findings suggestive of endomyocarditis or endomyocardial fibrosis were found in 31/60 patients (51.7%).

**Conclusions:**

Border zone distribution of cerebral ischemia without hemodynamic compromise is the most frequent imaging pattern in patients with HES, occurring in 2/3 of patients who develop ischemic stroke.

**Supplementary Information:**

The online version contains supplementary material available at 10.1007/s10072-022-06134-4.

## Introduction

Ischemic stroke is one of the most frequent causes of death and disability worldwide. The most important causes for ischemic stroke include cardioembolism, large vessel disease, and small vessel disease, but there are many additional less frequent causes which need to be investigated when stroke etiology remains unclear. Cerebral ischemia is a well-known complication of hypereosinophilic syndromes (HES) [1, 2]. HES are defined as clinical manifestations attributed to end-organ dysfunction associated with peripheral eosinophilia, which may occur in the setting of several conditions, such as parasitic infections, drug hypersensitivity, eosinophilic granulomatosis with polyangiitis, and other autoimmune diseases [3]. Additionally, HES may be caused by chronic eosinophilic leukemia, by myeloid or lymphocytic neoplasms associated with specific genetic rearrangements and may be idiopathic [4]. One of the reported mechanisms of ischemic stroke in patients with HES is cardioembolism, which involves the formation of thrombus in the left ventricle as a complication of eosinophilic endomyocarditis [5]. Other mechanisms appear to be involved, since non-cortical cerebral ischemic lesions in arterial border zones were also reported in several patients without hemodynamic compromise [6]. We report a patient with idiopathic HES and border zone cerebral ischemia. Furthermore, we present a systematic review of patients with HES and ischemic stroke focused on imaging patterns of cerebral ischemia.

## Case report

**Fig. 1:**
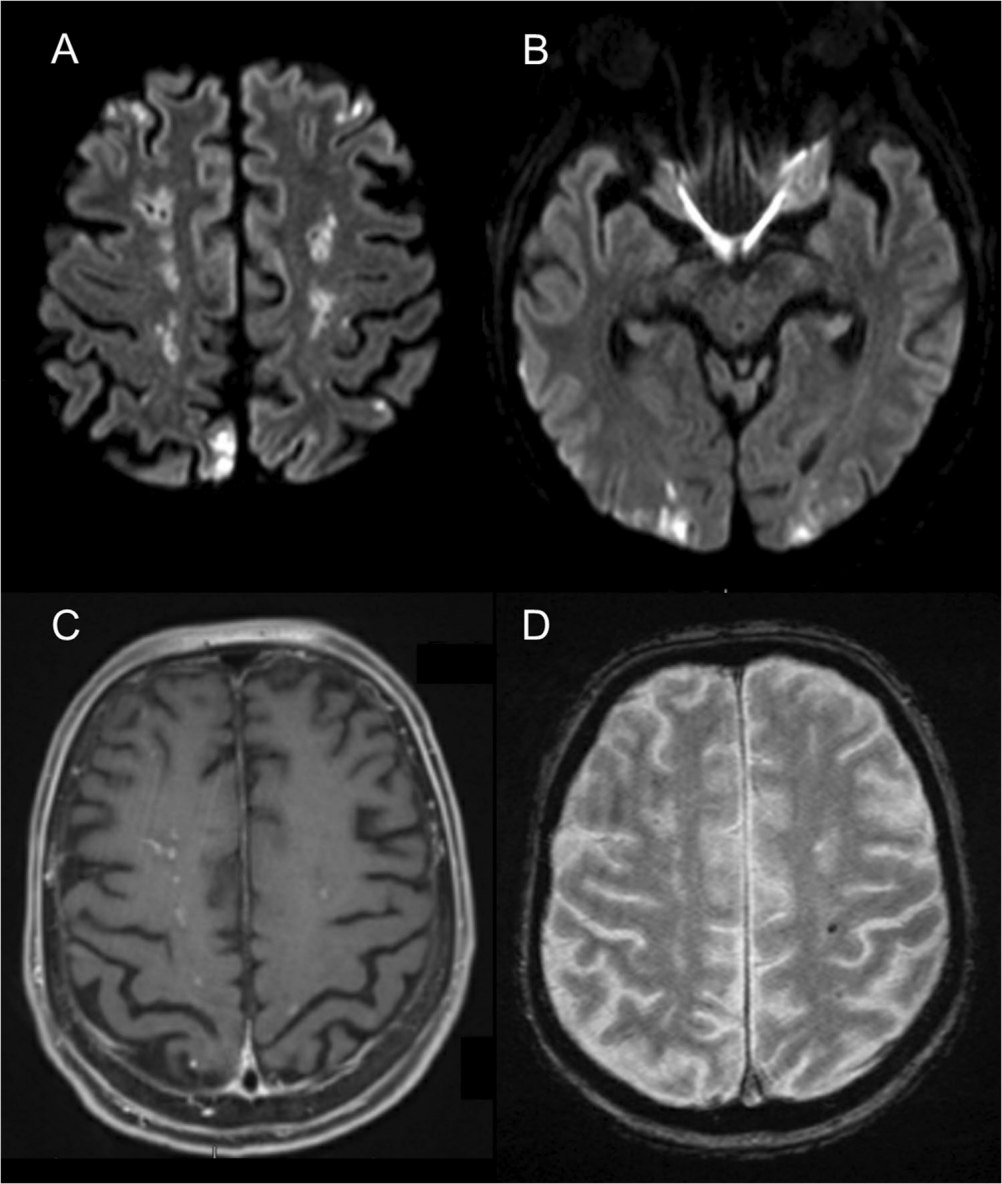
Brain magnetic resonance imaging showing multiple acute and subacute border zone infarcts in diffusion weighted imaging (**A**, **B**), some of which present gadolinium enhancement in T1 (**C**), and an example of a cerebral microbleed in T2-gradient echo (**D**)

A 60-year-old previously healthy man presented with a progressive gait disturbance and psychomotor slowing in the last 2 weeks. Initial neurological examination revealed reduced attention, bilateral ideomotor apraxia, and small-stepped gait. A relevant troponin-T increase (412 pg/mL) without electrocardiographic changes and a relative and absolute eosinophilia (27%, 2.2 × 10^9^/L) were found at admission. Initial cerebral magnetic resonance imaging (MRI) revealed multiple bilateral acute and subacute internal and external border zone infarctions, some of which with gadolinium enhancement, cortical infarctions, and several lobar microbleeds (Fig. 1). Duplex ultrasound and MR-angiography did not show any stenosis of the craniocervical arteries. Transthoracic and transesophageal echocardiography revealed no abnormalities. Cardiac MRI revealed diffuse subendocardial perfusion deficit and diffuse subendocardial late gadolinium enhancement compatible with endomyocarditis. After an extensive search for underlying infections, malignancies, and autoimmune diseases through comprehensive laboratorial workup, thoracic and abdominal computer tomography (CT), and bone marrow biopsy, an idiopathic HES was diagnosed.

## Systematic review

A systematic review was conducted in Pubmed using the search term [“hypereosinophilic syndrome” AND (stroke OR isch* OR infarct)] on November 6^th^ 2021 according to the PRISMA guidelines. This yielded 163 results and 20 additional papers were retrieved from the analysis of the references of relevant papers. We selected papers written in English, French, German, Portuguese, and Spanish, which reported adult patients with idiopathic HES and with HES associated with genetic rearrangements (namely FIP1L1-PDGFRA). Authors of papers which could not be retrieved or in which neuroimaging was not reported were contacted. From the initial title and abstract review of these 183 papers, 57 papers were excluded (no HES patient reported = 19, secondary HES = 16, language = 10, pediatric age = 8, review paper = 4). The full-text of the remaining 125 papers was reviewed and 85 were excluded (no ischemic stroke = 58, CT/MRI not available = 10, infection-related HES = 3, no access to full-text = 10, others = 5) (Supplementary Figure). Forty studies with a total of 63 patients and our additional case (*n* = 64) were included in the analysis.

Details of the included studies and patients can be found in Supplementary Table. Median age was 55 years (interquartile range 48–62), 31.3% were female (*n* = 20), and median absolute eosinophile count at diagnosis was 10.2 × 10^9^/L (interquartile range 4.4–18.9). A total of 61/64 patients underwent brain MRI and 39/64 underwent an angiographic exam. A border zone pattern of cerebral ischemic lesions was reported in 42 patients (65.6%), a single territorial or small peripheral cortical infarct was reported in 7 patients (10.9%), and 15 patients presented multiple acute embolic infarcts with no border zone distribution (23.4%). Sixty patients underwent transthoracic/transoesophageal echocardiography, and 22 underwent cardiac MRI: an intracardiac thrombus was reported in 15/60 patients (25%), and findings suggestive of endomyocarditis or endomyocardial fibrosis were found in 31/60 patients (51.7%). Angiographic findings suggestive of cerebral vasculitis were found in 1/39 patients; large vessel occlusion/stenosis was reported in 2/39 patients. Patients with border zone pattern of ischemia presented more frequently with encephalopathy (45.2% versus 13.6%, *p* = 0.011). There was no association between the presence of border zone pattern of ischemia and age (*p* = 0.323), sex (*p* = 0.272), absolute eosinophile count (*p* = 0.582), presence of endomyocarditis or endomyocardial fibrosis (*p* = 0.929), or presence of intracardiac thrombus (*p* = 0.423).

## Discussion

The main conclusion of our study is that among patients with HES who develop cerebral ischemia, approximately two thirds of patients present a border zone pattern of cerebral ischemia. As our case report illustrates, hemodynamic causes of ischemic stroke, such as an internal carotid artery or intracranial stenosis, are typically absent in HES patients with cerebral ischemia. The occurrence of vasculitis and large vessel occlusion is also not frequent. An intracardial thrombus was found in 25% of patients, which on its own does not explain the border zone distribution of ischemia. Several mechanisms were proposed to cause ischemia in HES patients, namely direct eosinophile-induced endothelial toxicity and subsequent local thrombogenicity [6], eosinophilic vasculitis [7], prothrombotic state and hypercoagulability [8], and/or cardiac microembolism [9]. An interesting mechanism which may explain the border zone pattern was proposed by Aida et al. [10]. It comprises the formation of microthrombi in the heart (related primarily to eosinophilic cardiac involvement and endomyocardial damage) or in the vessels (related primarily to a state of hypercoagulability), which can embolize or form in the cerebral circulation. When reaching the cerebral microcirculation, these microemboli will be directly washed out from areas with medium- and high-flow, and lodge in border zone areas which typically have low flow [11]. Experimental studies have indeed found that particles between 150 and 210 microns embolize preferentially in border zone areas [12] and autopsy studies also found microembolic occlusions of leptomeningeal arteries in patients with border zone infarcts [13]. In patients with HES, the state of hypercoagulability and possible microcirculation dysfunction induced by eosinophil-triggered endothelium toxicity may further contribute to this mechanism. There are two main types of distribution of border zone infarcts in the brain, and it has been suggested that internal border zone infarcts are more frequently associated with a hemodynamic mechanism, whereas cortical border zone infarcts are more frequently associated with embolic mechanisms [11]. Unfortunately, the specific distribution of border zone infarcts was not available for the majority of studies which we included in our systematic review. Because of the relatively low frequency of large vessel occlusion, it is expected that clinically severe strokes and need for emergent endovascular treatment [14] are infrequent in these patients. This was demonstrated in the study by Tennenbaum et al., in which 16 HES patients with stroke presented with a median National Institutes of Health Stroke Scale of 4 [6].

In conclusion, our study demonstrates that border zone cerebral ischemia in patients with HES and ischemic stroke is frequent, and possible underlying mechanisms include impaired washout of the microemboli from the cerebral microcirculation.

## Supplementary Information

Below is the link to the electronic supplementary material.

Supplementary file1 (DOCX 64 KB)
