# Supplementary material for: Imaging patterns of cerebral ischemia in hypereosinophilic syndrome: case report and systematic review
Source: Neurol Sci. 2022 May 19;43(8):5091–4. doi: 10.1007/s10072-022-06134-4 (PMC9349058; doi:10.1007/s10072-022-06134-4)
Supplement: Supplementary file 1 — Supplementary file1 (DOCX 64 KB) [file 10072_2022_6134_MOESM1_ESM.docx]

**Supplementary Figure**: PRISMA flow diagram

**Identification of studies via databases**

Records removed before screening: n = 0

Records identified from:

Databases (n = 163)

Review of the references of relevant papers (n = 20)

**Identification**

Records excluded (n = 57)

* no HES patient reported (n = 19)

* secondary HES (n = 16)

* language criteria (n = 10)

* pediatric age (n = 8)

* review paper (n = 4)

Records screened

(n = 183)

**Screening**

**Included**

Reports sought for retrieval

(n = 126)

Reports not retrieved

(n = 10)

Reports assessed for eligibility

(n = 116)

Reports excluded (n=75)

* no ischemic stroke (n = 58)

* neuroimaging not available (n = 10)

* infection-related HES (n=3)

* no HES patient reported (n = 3)

* pediatric age (n = 1)

* peri-interventional iatrogenic stroke (n = 1)

Studies included in review (n = 40)

Patients included in the study (n=64)

* cases collected from reports (n = 63)

* local case (n = 1)

Supplementary Table: Baseline characteristics of patients with hypereosinophilic syndrome included in the study

| **STUDY** | **Number of patients** | **Sex** | **Age (years)^*^** | **Eosinophile count (x10^9^/L)^*^** | **Imaging pattern of cerebral ischemia** | | | **Angiographic findings** | **Intracardiac thrombus** | **Endomyocarditis / endomyocardial fibrosis** |
| --- | --- | --- | --- | --- | --- | --- | --- | --- | --- | --- |
|  |  |  |  |  | Border zone infarcts | Single infarct | Multiple infarcts, no border zone pattern |  |  |  |
| **Otto et al, 1993 [1]** | 1 | Male | 43 | 11.5 | 🗹 | 🗷 | 🗹 | NA | 🗷 | 🗷 |
| **Kwon et al, 2001 [2]** | 3 | Male (3) | 55 | 4.7 | 3/3 | 0/3 | 0/3 | Normal | 1/3 | 2/3 |
| **Sarazin et al, 2004 [3]** | 1 | Male | 51 | 26 | 🗹 | 🗷 | 🗷 | NA | 🗷 | 🗹 |
| **Engelmann et al, 2004 [4]** | 1 | Male | 40 | 17 | 🗷 | 🗷 | 🗹 | NA | 🗹 | 🗹 |
| **Rojas et al, 2006 [5]** | 1 | Male | 70 | 10.6 | 🗹 | 🗷 | 🗷 | Normal | 🗷 | 🗷 |
| **Zürn et al, 2006 [6]** | 1 | Female | 31 | 4.7 | 🗹 | 🗷 | 🗷 | NA | 🗷 | 🗹 |
| **Fazel et al, 2009 [7]** | 1 | Male | 64 | 6.1 | 🗹 | 🗷 | 🗷 | Normal | 🗹 | 🗹 |
| **Perini et al, 2009 [8]** | 1 | Female | 63 | 118.4 | 🗹 | 🗷 | 🗷 | NA | 🗷 | 🗹 |
| **Lee et al, 2009 [9]** | 1 | Male | 52 | 5.5 | 🗹 | 🗷 | 🗷 | Normal | 🗷 | 🗷 |
| **Kono et al, 2009 [10]** | 1 | Male | 79 | 23.2 | 🗹 | 🗷 | 🗷 | Normal | 🗷 | 🗷 |
| **Grigoryan et al, 2009 [11]** | 1 | Male | 48 | 4.1 | 🗹 | 🗷 | 🗷 | NA | 🗷 | 🗷 |
| **Ahn et al, 2010 [12]** | 1 | Male | 56 | 9.4 | 🗹 | 🗷 | 🗷 | Normal | 🗷 | 🗹 |
| **Takeuchi et al, 2010 [13]** | 1 | Female | 23 | 168 | 🗷 | 🗹 | 🗷 | LVO | 🗷 | 🗷 |
| **Sethi et al, 2010 [14]** | 3 | Male (2)  Female (1) | 48 | 31.4 | 2/3 | 0/3 | 1/3 | Normal (1/3), NA (2/3) | 0/3 | 0/3 |
| **Dujardin et al, 2011 [15]** | 1 | Male | 56 | 3.5 | 🗹 | 🗷 | 🗷 | NA | 🗷 | 🗷 |
| **van Gaalen et al, 2011 [16]** | 1 | Female | 18 | 3.9 | 🗷 | 🗹 | 🗷 | PICA dissection | 🗷 | 🗷 |
| **Wise et al, 2013 [17]** | 1 | Male | 66 | 5.1 | 🗷 | 🗷 | 🗹 | Normal | 🗷 | 🗷 |
| **Wang et al, 2013 [18]** | 1 | Male | 56 | 14.5 | 🗷 | 🗷 | 🗹 | Normal | 🗹 | 🗷 |
| **Yhim et al, 2013 [19]** | 1 | Male | 51 | 40.4 | 🗷 | 🗷 | 🗹 | NA | 🗹 | 🗹 |
| **Aida et al, 2013 [20]** | 4 | Male (2)  Female (2) | 51 | 5.3 | 3/4 | 0/4 | 1/4 | Normal (3/4), NA (1/4) | 1/4 | 3/4 |
| **Khwaja et al, 2013 [21]** | 1 | Male | 68 | 42.5 | 🗹 | 🗷 | 🗷 | NA | 🗹 | 🗷 |
| **Wu et al, 2014 [22]** | 1 | Male | 62 | 4.8 | 🗹 | 🗷 | 🗷 | Normal | 🗷 | 🗷 |
| **Lai et al, 2015 [23]** | 1 | Male | 52 | 16.6 | 🗹 | 🗷 | 🗷 | NA | 🗹 | 🗹 |
| **Grolla et al, 2015 [24]** | 1 | Male | 48 | NA | 🗷 | 🗷 | 🗹 | NA | 🗹 | 🗷 |
| **Marton et al, 2015 [25]** | 1 | Male | 33 | 8.5 | 🗹 | 🗷 | 🗷 | NA | 🗷 | 🗹 |
| **Chen et al, 2017 [26]** | 2 | Male (1)  Female (1) | 41 | 5.2 | 🗷 | 1/2 | 1/2 | Normal | NA | NA |
| **Chalayer et al, 2017 [27]** | 1 | Male | 26 | 20 | 🗹 | 🗷 | 🗷 | NA | 🗷 | 🗹 |
| **Wang et al, 2017 [28]** | 1 | Female | 48 | 3.2 | 🗷 | 🗷 | 🗹 | NA | 🗷 | 🗹 |
| **Lee et al, 2017 [29]** | 1 | Male | 30 | 26.4 | 🗷 | 🗹 | 🗷 | NA | 🗹 | 🗹 |
| **Mulroy et al, 2018 [30]** | 1 | Female | 27 | NA | 🗹 | 🗷 | 🗷 | NA | NA | NA |
| **Curras-Martin et al, 2019 [31]** | 1 | Male | 63 | 16.2 | 🗷 | 🗷 | 🗹 | NA | 🗹 | 🗹 |
| **Chiu et al, 2019 [32]** | 1 | Female | 63 | 12.2 | 🗹 | 🗷 | 🗷 | NA | 🗷 | 🗹 |
| **Wasilewski, 2019 [33]** | 1 | Female | 59 | 179.5 | 🗹 | 🗷 | 🗷 | NA | NA | 🗷 |
| **Chua et al, 2020 [34]** | 1 | Female | 65 | 12 | 🗷 | 🗷 | 🗹 | Normal | 🗷 | 🗷 |
| **Lambird et al, 2019 [35]** | 1 | Male | 62 | 11.8 | 🗷 | 🗷 | 🗹 | NA | 🗷 | 🗹 |
| **Li et al, 2020 [36]** | 1 | Female | 55 | 5.3 | 🗷 | 🗷 | 🗹 | LVO; bilateral MCA wall thickening and enhancement | 🗷 | 🗷 |
| **Demetriades et al, 2020 [37]** | 1 | Male | 57 | 13 | 🗹 | 🗷 | 🗷 | NA | 🗹 | 🗹 |
| **Hwang et al, 2021 [38]** | 1 | Male | 55 | 13.3 | 🗹 | 🗷 | 🗷 | NA | 🗹 | 🗷 |
| **Barbind et al, 2021 [39]** | 1 | Female | 65 | 68 | 🗹 | 🗷 | 🗷 | Normal | 🗹 | 🗷 |
| **Tennenbaum et al, 2021 [40]** | 16 | NA | 51 | 12.1 | 11/16 | 2/16 | 2/16 | Normal (16/16) | 1/16 | 8/16 |
| **Present case** | 1 | Male | 60 | 2.2 | 🗹 | 🗷 | 🗷 | Normal | 🗷 | 🗹 |

NA: not available / not reported. LVO: large vessel occlusion. PICA: posterior inferior cerebellar artery. MCA: middle cerebral artery.

* Values presented as mean if >1 patient reported

1. Otto V. [Neurologic complications in hypereosinophilia syndrome--a case report]. Fortschr Neurol Psychiatr 1993;61:310-2.

2. Kwon SU, Kim JC, Kim JS. Sequential magnetic resonance imaging findings in hypereosinophilia-induced encephalopathy. J Neurol 2001;248:279-284.

3. Sarazin M, Caumes E, Cohen A, Amarenco P. Multiple microembolic borderzone brain infarctions and endomyocardial fibrosis in idiopathic hypereosinophilic syndrome and Schistosoma mansoni infection. J Neurol Neurosurg Neuropsychiatry 2004;75:305-307.

4. Engelmann MG, Kolbe T, Faul C, Steinbeck G. Hypereosinophilic syndrome associated with heterozygous factor V gene mutation: an unusual combination resulting in an acute coronary syndrome and recurrent cerebral stroke-a case report. Angiology 2004;55:221-225.

5. Rojas GJ, Castro DM, Vigo-Guevara GL, Ferrua M, Barriga-Maldonado V, Rotta-Escalante R. [Hypereosinophilic encephalopathy with multiple cerebral infarctions in neighbouring vascular territories associated with prostate cancer]. Rev Neurol 2006;43:762-764.

6. Zürn CS, Brehm BR, Rüb N, et al. [Recurrent transient ischaemic attacks in a patient with pansinusitis]. Internist (Berl.) 2006;47:1165-1171.

7. Fazel R, Dhaliwal G, Saint S, Nallamothu BK. Clinic problem-solving. A red flag. N Eng J Med 2009;360:2005-2010.

8. Perini GF, Kassab C, Bley C, Monzillo PH, Thomaz RB, Hamerschlak N. Acute cerebral infarction in watershed distribution in a patient with hypereosinophilic syndrome. Arq Neuropsiquiatr 2009;67:510-512.

9. Lee EU, Lee YJ, Lee SR, Park DW, Kim HY. Hypereosinophilia with multiple thromboembolic cerebral infarcts and focal intracerebral hemorrhage. Korean J Radiol 2009;10:511-514.

10. Kono Y, Itoh Y. Diffusion-weighted imaging of encephalopathy related to idiopathic hypereosinophilic syndrome. Clin Neurol Neurosurg 2009;111:551-553.

11. Grigoryan M, Geisler SD, Louis EK, Baumbach GL, Davis PH. Cerebral arteriolar thromboembolism in idiopathic hypereosinophilic syndrome. Arch Neurol 2009;66:528-531.

12. Ahn SW, Han MK. Multiple bilateral cerebral infarcts in a patient with idiopathic hypereosinophilic syndrome. Neurol India 2010;58:793-794.

13. Takeuchi S, Takasato Y, Masaoka H, et al. Middle cerebral artery occlusion resulting from hypereosinophilic syndrome. J Clin Neurosci 2010;17:377-378.

14. Sethi HS, Schmidley JW. Cerebral infarcts in the setting of eosinophilia: three cases and a discussion. Arch Neurol 2010;67:1275-1277.

15. Dujardin S, Schots R, De Raedt S. Suspected "t-cell-mediated" hypereosinophilic syndrome presenting with cerebral watershed infarcts. Case Rep Neurol Med 2011;2011: 834308.

16. van Gaalen J, van Dijk EJ, van Deuren M, de Leeuw FE. Dissection of the posterior inferior cerebellar artery in the hypereosinophilic syndrome. J Neurol 2011;258:2278-2280.

17. Wise FM, Olver JH. A 66-year-old man with multiple cerebral and cerebellar infarcts due to idiopathic hypereosinophilic syndrome. J Clin Neurosci 2013;20:1442-1443.

18. Wang S, Wang A, Guo B, Zhu S, Chi Z, Zhao X. Löffler endocarditis with multiple cerebral embolism. J Stroke Cerebrovasc Dis 2014;23:1709-1712.

19. Yhim HY, Lee SR. Rapid left ventricular thickening and thrombus caused by FIP1L1/PDGFRA-negative hypereosinophilic syndrome. Tex Heart Inst J 2013;40:362-363.

20. Aida L, Parkhutik V, Tembl JI, Martín N, Frasquet M, Bataller L. Embolism and impaired washout: a possible explanation of border zone strokes in hypereosinophilic syndrome. J Neurol Sci 203;325:162-164.

21. Khwaja GA, Duggal A, Kulkarni A, et al. Hypereosinophilia – an unusual cause of multiple embolic strokes and multiorgan dysfunction. J Clin Diagn Res 2013;7:2316-2318.

22. Wu X, Guo Y, Tan X. Acute cerebral infarction in watershed distribution in a patient with hypereosinophilic syndrome without cardiac lesion. Neurol Sci 2014;35:1607-1610.

23. Lai CH, Chang SL, Lin WW, Hsiung MC, Juan YH, Wang TL. Atypical presentation of intracardiac floating thrombi in hypereosinophilic syndrome complicated with stroke and systemic embolization: a case report. Medicine (Baltimore) 2015;94:e1844.

24. Grolla E, Vestra MD, Bonanni L, Cutolo A, Rigo F. A rare case of aortic valve thrombosis in patient with idiopathic hypereosinophilic syndrome. Case Rep Cardiol 2015;2015:607107.

25. Marton I, Pósfai É, Annus JK, et al. Watershed infarction in hypereosinophilic syndrome: a diagnostic dilemma in FIP1L1-PDGFR alpha-associated myeloid neoplasm. Ideggyogy Sz 2015;68:212-216.

26. Chen H, Raza HK, Jing J, et al. Hypereosinophilic syndrome with central nervous system involvement: two case reports and literature review. Brain Inj 2017;31:1695-1700.

27. Chalayer E, Pelissier A, Tardy B. When hypereosinophilia leads to stroke. Eur J Case Rep Intern Med 2017;4:000614.

28. Wang H, Erban JK. Rapid change in mental status in a patient with hypereosinophilia. Case Rep Hematol 2017:2017:6936709.

29. Lee S, Choi CU, Kim EJ, Na JO. Regression of biventricular Loeffler’s endocarditis after early treatment. Eur Heart J Cardiovasc Imaging 2017;18:610.

30. Mulroy E, Cleland J, Anderson NE. Crescentic splinter haemorrhages reflect stroke pathophysiology in hypereosinophilic syndrome. Australas J Dermatol 2018;59:e211-e212.

31. Curras-Martin D, Patel S, Qaisar H, et al. Acute kidney injury secondary to thrombotic microangiopathy associated with idiopathic hypereosinophilic syndrome: a case report and review of the literature. J Med Case Rep 2019;13:281.

32. Chiu MH, Sharma NC. Myocarditis and eosinophilia: three cases of hypereosinophilic syndrome and myocarditis. CJC Open 2019;1:100-102.

33. Wasilewski A. Teaching NeuroImages: Multifocal cerebral infarcts as a presentation of idiopathic hypereosinophilic syndrome. Neurology 2019;92:e2178.

34. Chua CE, Ling V, Jing M, et al. An unusual presentation of idiopathic hypereosinophilic syndrome. J Thromb Thrombolysis 2020;50:473-476.

35. Lambird E, Patel D, Amble A, Henderson E, Muddassir S. A rare case of hypereosinophilic syndrome-induced shower thrombus responsive to nilotinib. Cureus 2020;12:e8341.

36. Li QF, Zhang Q, Huang YF, Zhang ZX. Acute progressive stroke with middle cerebral artery occlusion caused by hypereosinophilic syndrome: a case report. BMC Neurol 2020;20:361.

37. Demetriades P, Speke L, Wilson L, Khan JN. When multimodality cardiac imaging saves the day: rare cause of embolic strokes. BMJ Case Rep 2020;13:e232786.

38. Hwang JW, Kim H, Cho SW, et al. Idiopathic hypereosinophilic syndrome with intracardiac atypical linear-shaped and floating thrombus presenting as embolic cerebral infarction. J Cardiol Cases 2021;23:193-197.

39. Barbind KL, Boddu R, Shijith KP, Mishra K. Hypereosinophilia: a rare cause of stroke and multiorgan dysfunction. BMJ Case Rep 2021;14:e242619.

40. Tennenbaum J, Groh M, Venditti L, et al. FIP1L1-PDGFRA-associated hypereosinophilic syndrome as a treatable cause of watershed infarction. Stroke 2021;52:e605-e609.
